# Use of the Baby-Led Weaning (BLW) Method in Complementary Feeding of the Infant—A Cross-Sectional Study of Mothers Using and Not Using the BLW Method

**DOI:** 10.3390/nu14122372

**Published:** 2022-06-08

**Authors:** Agnieszka Białek-Dratwa, Monika Soczewka, Mateusz Grajek, Elżbieta Szczepańska, Oskar Kowalski

**Affiliations:** 1 Department of Human Nutrition, Department of Dietetics, Faculty of Health Sciences in Bytom, Medical University of Silesia in Katowice, ul. Jordana 19, 41-808 Zabrze, Poland; eszczepanska@sum.edu.pl (E.S.); okowalski@sum.edu.pl (O.K.); 2 Department of Pediatric Diabetes, Auxology and Obesity, Poznan University of Medical Sciences, 60-512 Poznan, Poland; monika.soczewka@student.ump.edu.pl; 3 Doctoral School, Poznan University of Medical Sciences, Fredry St. 10, 61-701 Poznan, Poland; 4 Department of Public Health, Department of Public Health Policy, Faculty of Health Sciences in Bytom, Medical University of Silesia in Katowice, ul. Piekarska 18, 41-902 Bytom, Poland; mgrajek@sum.edu.pl

**Keywords:** child nutrition, expanding the diet of infants, BLW, complementary feeding

## Abstract

Baby-led weaning (BLW) is an increasingly popular way of expanding a baby’s diet. It is based on the baby becoming physically ready to feed himself, effectively supplementing his diet, which until now has been based on breast milk or modified milk. The aim of the study was to assess mothers’ knowledge about the use of the BLW method to expand the diet of a young child. The essence of the study assumed the analysis of the advantages and disadvantages of using this method indicated by mothers. Materials and Methods: A total of 320 mothers participated in the study. Data for the study were collected anonymously using the CAWI method. The research tool was the original questionnaire relating to the knowledge about the BLW method and the application of the BLW method in practice. Results: The BLW method was used by 240 (75%) women. The reasons for not using the BLW method were: the child did not cooperate n = 30 (37.5%) and was not ready to use the BLW method n = 20 (25%). In total, 182 (75.8%) mothers using BLW and 63 (78.8%) mothers not using BLW started extending the diet before the child was 6 months old. According to 270 (84.4%) mothers, including 205 (85.4%) using BLW, stable sitting in a highchair/on the lap is a decisive factor for starting the dietary expansion with the BLW method. Conclusions: Mothers’ knowledge of the BLW method as a way of expanding a young child’s diet was insufficient. It seems important to implement appropriate educational activities on the methods of expanding children’s diets to broaden parents’ knowledge of the influence of nutrition on infant development.

## 1. Introduction

Baby-led weaning (BLW) is an increasingly popular way of expanding an infant’s diet [1,2]. It is based on the baby becoming physically ready to eat on his or her own, effectively supplementing his or her diet, which was previously based on breast milk or modified milk [3]. In the traditional approach to complementary feeding, parents usually feed their infants pureed foods (mush) with a spoon, gradually introducing an increasing variety of tastes and textures as they grow, until solid foods are introduced [4,5]. The process of diet expansion with the BLW method is guided by the child, using its skills and instinct. According to Brown and Lee [6], BLW is “a procedure in which the infant feeds himself, and feeding by the parent or serving smooth purees may occur occasionally, up to 10% of the total feeding time”. The role of the first solid foods, often referred to as complementary foods, in expanding an infant’s diet is not to replace breast milk or formula milk, but to be in addition to it [1].

An important issue in feeding the baby is the mother’s control over the baby’s feeding. Due to the development of appetite, it is beneficial to feed the child according to its needs [3]. The recommendations of the Polish Society for Paediatric Gastroenterology, Hepatology, and Nutrition (PTGHiŻD) [7] and the European Society for Paediatric Gastroenterology Hepatology and Nutrition (ESPGHAN) [5] take into account the fact that the child should decide whether to eat food and in what amount and the parents decide what the child should eat [8]. Parental attitudes such as forbidding, urging, coercing and other similar reactions cause defensive behavior in the child and are often the cause of feeding problems. Brown and Lee [6] pointed out that mothers who used BLW ensure that eating is a pleasure for both them and the child. These women showed less controlling behavior and approached the expansion of the infant’s diet much more calmly than mothers who spoon-fed [6,8,9,10,11].

### 1.1. Strengths of BLW

For children, the opportunity to eat by themselves has many benefits, not only nutritional. The opportunity for the child to eat on his or her own strengthens his or her development and draws his or her attention to the variety of food on offer rather than the person serving the food. Children who have the opportunity to eat freely not only improve their feeding skills in the nutritional sense but also develop the precision of grasping the products, and motor coordination, become active participants in the meal, and are involved in the full process, in contrast to infants who do not have the opportunity to reach for food independently and become an inactive child during the feeding process [12,13,14,15].

Using BLW the family sits down at the table together. The child is not fed while eating dinner with its parents so that the parents’ dinner does not become cold. For the most part, you can offer the child the same as the rest of the family. However, care should be taken that the products are not rich in salt, sugar, or saturated fatty acids [14]. In the BLW method, the infant dictates the pace of eating and what it wants to eat and what it does not want to eat. The infant treats eating meals as a kind of play, thanks to which it can imitate the behavior of people present at the table. They learn to share, wait for their turn, they watch carefully how different foods are eaten [1]. According to a study by D’Auria et al. [15], positive relationships within the family are deepened from the start, although they do not necessarily improve the family’s eating style. For many mothers, the fear of using the BLW method is the difficulty in serving a meal to a child outside the home, including the inability to warm up the food. However, with BLW this fear is unfounded. Restaurants have extensive menus, so there will be something the baby can eat. This is because children who eat using the BLW method are more curious about new tastes. Additionally, they are more willing to try eating something new. They pay attention to the difference between homemade food and food from a restaurant, for example. Therefore, such food will smell, look and taste differently, and additionally it will be brought to them only after some time. This is a new experience for children [1].

Children who were fed according to the principles of the BLW method at the time of introduction of complementary foods, according to the study ate healthy meals with their families and had less predisposition to bad eating habits in the future. An important factor is the dietary practices used by parents. If the parents eat properly and feed the child with such food, this way the infants, as adults, lead a healthier lifestyle and are more open to different culinary experiences from the beginning [16]. Eating habits formed in childhood often remain for life. When expanding the infant’s diet, the child should be allowed to eat at its own pace, not forced to finish the meal when we feel like it. If we let the child control its appetite, and eat according to its appetite, there is a high probability that it will not overeat [17]. This fact can be considered an important aspect of obesity prevention. Healthy eating practices from early childhood can also protect against other diseases the background of faulty nutrition, not only obesity but also diabetes or metabolic syndrome [18,19,20].

### 1.2. Weaknesses of BLW

One of the most frequently mentioned fears of parents in the use of BLW is the possibility of choking the child, which is connected with giving bigger pieces of food. Scientific studies show that infants from the age of 6–7 months have the developmental capacity to eat a variety of solids. The avoidance of solid food may in the future result in food refusal or selective eating. It has been proved that frequent administration of meals requiring biting and chewing pays off in the acceptance of particular foods and dishes and supports the development of the speech apparatus [18,21,22,23]. The American Academy of Pediatrics (AAP) recommends avoiding round and small products with a smooth and hard surface and a cross-section similar to the shape of the child’s airway [24]. Foods that are most likely to cause choking include: sausages hard candies, seeds and nuts (whole), raw apples and carrots, chewing gums, and roasted corn. Non-food items that are most likely to choke a child are usually: plastic bags and balloons, and small and round toys [25]. Avoidance of the above foods reduces the risk of choking. The AAP includes eating while moving and during other activities, when the child is not focusing attention on chewing, among the circumstances conducive to choking in a group of healthy children [24]. Choking is a vomiting reflex that allows the removal of food fragments from the airway when they prove too large to swallow. In adults, this reflex starts from the back of the tongue. In children, on the other hand, this reflex is activated in a different part of the tongue—i.e., closer to the anterior part of the tongue. Therefore, in children, this fact makes it easier to trigger. The reflex itself is part of the body’s defensive reaction, but it is rarely associated with a choking hazard. It occurs sporadically and is certainly not a danger for the child because a piece of food is spat out before it reaches the throat [1]. The majority of infants develop the ability to eat solid food between 17 and 26 weeks of age. They develop the ability to sit with support and acquire neuromuscular maturity that allows them to control head and neck movements to eat from a spoon. At this time, the reflex to remove foreign bodies from the mouth, which made feeding with food other than liquid difficult, ceases [5].

Children who use the BLW method are sometimes exposed to a variety of food products and foods eaten by the family, including ready-to-eat foods, which can lead to the appearance of allergic reactions to food. In the future, children who consume heavily processed food may be at greater risk of developing several diseases resulting from poor nutrition, including type 2 diabetes, as well as obesity and metabolic syndrome, which are increasingly common among younger people. Children fed using the BLW method are exposed to many risks, therefore further research is needed on feeding children using the BLW method [1]. Morison et al. [26] analyzed the diet of infants aged 6–8 months who were fed using the BLW method (*n* = 25) or traditionally, with a spoon (*n* = 26). Despite similar energy intakes, BLW infants consumed less protein, carbohydrate, and fiber and more fat (including saturated fat). Lower intakes of iron, zinc, calcium, vitamin B12 and vitamin C were also observed, although there was no apparent difference in sodium intake. It has not yet been established whether these differences persist at older ages, which would be relevant to the method of infant dietary expansion used.

The main aim of the study was to assess the knowledge of mothers on the use of the BLW method as a way of expanding the diet of a young child and to compare the knowledge of mothers using and not using the BLW method. The essence of the study also assumed the analysis of the advantages and disadvantages of using this method indicated by mothers in terms of practical use of BLW in the process of expanding the diet of infants.

## 2. Materials and Methods

### 2.1. Study Group

In designing the study, we calculated the minimum sample size on the basis of data from the Polish Central Statistical Office (GUS) on the size of the population of mothers of children aged 0-36 months about 1,133,000 born children, the assumed confidence level for the results at the level of 0.95, the size of the fraction of 0.3, and the assumed maximum error of 0.05. Calculating the minimum sample size, we obtained the result of 323 as the required number of people in the study.

A total of 347 mothers consented to the study. In total, 332 mothers completed the questionnaire correctly. After considering the inclusion and exclusion criteria, 320 mothers were included in the final analysis of the study. Data for the study were collected anonymously using the CAWI method (Computer-Assisted Web Interview). The internet questionnaire was distributed on forums and discussion groups designed for mothers. All participants were informed about the aim of the study and the method of data sharing, voluntary participation in the study, and its anonymity, and agreed to participate in the study.

The study was conducted between February and May 2020. Information collected from 320 women was considered for the final data analysis, considering the inclusion and exclusion criteria of the study.

The study did not require the authors to obtain approval from a bioethics committee in light of the Act on Physician and Dentist Professions of 5 December 1996, which includes a definition of medical experimentation. The study was by the Declaration of Helsinki.

#### Rationale for Selecting the Group

According to the current Polish law, after giving birth, a mother is entitled to a maternity leave of 20 weeks for one child, 31 weeks for twins, 33 weeks for three children, 35 weeks for four children, and 37 weeks for five and more children—regardless of the number of older children in the family [27]. After this time, parental leave can be taken, which entitles both parents to take it. Parental leave lasts 32 weeks in the case of the birth of 1 child. However, in practice, the majority of those who stay at home with the child after birth are mothers. In 2019, from the data of the Ministry of Family, Labour and Social Policy in Poland, 367,800 women, and 12,000 men took advantage of maternity leave [28]. According to the Polish Social Insurance Institution, in 2021, 99% of women and only 1% of men took parental leave. By the end of August 2021, out of 302,700 people who received a parental benefit, men only comprised 2700 [29]. Therefore, mothers were invited to the study on infant dietary expansion, as they are the ones who mostly spend time with their children and are responsible for expanding their children’s diet. The study took into account children aged 0–36 months because it is in this group that dietary expansion begins; children in Poland at this age usually remain at home with their mother until the age of one due to maternity and parental leave due to parents. Additionally, in Poland, until the child is 36 months old, children attend a childcare facility such as a nursery. From the age of 36 months (3 years), children start their early childhood education in a kindergarten.

### 2.2. Inclusion and Exclusion Criteria

The inclusion criteria were: female sex, having a child aged 0 to 36 months, knowledge of the BLW method, consent to the study, and correct and complete completion of the questionnaire.

Criteria for exclusion from the study: male gender, age of the child over 36 months or not having a child, unfamiliarity with the BLW method, lack of consent to participate in the study, incorrectly completed questionnaire, including failure to answer the questions.

### 2.3. Research Tool

The research tool was the author’s questionnaire containing a summary of 29 questions: 10 questions concerning sociodemographic data of the mother (age, place of residence, education, occupational activity, material status, number of children) and the child (child’s sex, child age). The questionnaire contained 19 questions concerning the knowledge of the BLW method and the application of the BLW method in practice—including the knowledge of the BLW method, the fact of using the BLW method, the reason for using the BLW method and not using it, the fact of breastfeeding during dietary expansion with the BLW method, differences between the traditional dietary expansion and the BLW method, the age when one can start the expansion of an infant’s diet, advantages and disadvantages of the BLW method, the method of using the BLW method, the choice of food products used in the BLW method. The questionnaire consisted of closed questions, single and multiple-choice, and open questions requiring an answer in 2–3 sentences, which at the same time were checking questions.

Before the actual study, a pilot study was conducted. It was conducted twice among the same group of 20 mothers with a one-month interval to avoid the freshness effect. The women surveyed had no comments on the questionnaire and the results obtained in the two surveys were reproducible. To assess the reproducibility of the results obtained by the questionnaire used, the ϰ (Kappa) parameter was calculated for each question of the questionnaire (results obtained in the pilot study and after one month)—for 71.5% of the questions a very good (ϰ ≥ 0.80) concordance of answers were obtained and for 19.2% of the questions a good (0.79 ≥ ϰ ≥ 0.60) concordance of methods was obtained. Only for 9.3% of the questions in the questionnaire analyzed, the concordance between the results obtained in the baseline and repeat test was moderate (ϰ < 0.59). Cronbach’s α coefficient for the standardization sample was 0.91, which indicates the high reliability of the selected questions.

### 2.4. Statistical Analysis

For the analysis of the collected data the software programs were used: Microsoft Office Word, Microsoft Office Excel, and Statistica 13.0. χ^2^ test was used for statistical analysis and s, the level of significance was assumed to be α, *p* < 0.05. Cramer’s V coefficient value was calculated for the level of significance assumed to be α, *p* < 0.05.

## 3. Results

### 3.1. Characteristics of the Study Group

The study was conducted among 320 mothers of children aged 0 to 36 months. Participating women were predominantly aged 19–30 years (*n* = 169; 52.8%), lived in urban areas (*n* = 253; 79.1%), had higher education (*n* = 265; 82.8%), were on maternity leave, parental leave, sick leave or not working at the time of the study (*n* = 214; 66.9%), and had one child (*n* = 222; 69.4%). The children of the women surveyed were most often aged: 7–12 months (*n* = 114; 35.6%) and 12–24 months (*n* = 112; 35.0%) (Table 1).

### 3.2. Use and Knowledge of BLW by the Surveyed Group of Mothers

Out of 320 mothers participating in the study, 240 (75.0%) used the BLW method. Among the reasons why mothers did not use the BLW method, the most frequent were: the child did not cooperate (*n* = 30; 37.5%), was not ready to use the BLW method (*n* = 20; 25%), and in mothers’ opinion, BLW is not a suitable method of expanding the diet of a young child (*n* = 15; 18.75%) (Table 2).

Of the mothers using the BLW method, 217 (90.4%) thought that its principles were consistent with the recommendations for expanding the diet of a young child. However, among all the women participating in the study 41 (12.8%) were unable to determine whether the principles of the BLW method were consistent with the dietary recommendations currently in force in Poland issued by PTGHiŻD [7]. The calculated value of Cramer’s V coefficient for the assumed significance level is 0.31 (low strength of dependence). The observed differences between the group of mothers using BLW and not using BLW in their answers are statistically significant.

Of all women, 318 (99.4%) correctly believed that breastfeeding should not be abandoned during BLW use, including a similar proportion of BLW users and non-users (Table 3). The observed differences in answers given by mothers using BLW and not using BLW are not statistically significant (*p* = 0.413).

The analysis of the obtained results showed that 245 (76.6%) women, including 182 (75.8%) mothers using BLW and 63 (75.8%) mothers not using BLW, started the dietary expansion of their child above the age of 6.0 months (Table 4). 

According to 270 (84.4%) mothers, including 205 (85.4%) practicing BLW, stable sitting in a highchair/on one’s lap is a decisive factor for the start of diet expansion with the BLW method. On the other hand, 247 (77.2%) mothers, including 194 (80.8%) mothers practicing BLW considered that the child must be at least 6 months old to be able to give products supplementing the diet. The doctor’s opinion was important for 44 (13.8%) women (Table 5).

The mothers’ opinion on the way of proceeding while using the BLW method was also examined. A total of 188 (58.8%) mothers, including 147 (61.3%) using BLW, considered the correct answer to give several products to the child to choose and eat what it wants. Further 82 (25.6%) women indicated the answer indicating the pace of diet expansion directed by the child and 48 (15.0%) indicated giving pieces of solid food as the most important activity in BLW practice. The calculated value of Cramer’s V coefficient for the adopted level of significance α, *p* = 0.002 is 0.22 (low strength of the relationship). The observed differences in answers given by mothers using BLW and not using BLW are statistically significant.

### 3.3. Disadvantages of BLW and Their Importance among the Surveyed Group

Table 6 and Table 7 present the results regarding the disadvantages of using BLW. According to 133 (41.6%) women, including 88 (36.7%) mothers using BLW, the risk of choking was the most significant disadvantage of BLW. Nutritional deficiency is a significant disadvantage for 119 (37.2%) women, including 35 (43.8%) mothers not using BLW. The need to spend more time was not a disadvantage according to 133 (55.4%) mothers using the BLW method.

For 220 mothers (68,8% 220/320), including 168 (70.0%) mothers using BLW and 52 (65.0%) mothers not using BLW, the regularity of serving additional food was significant. 

In answer to the question of whether the child can eat products from the family table, 220 (91.7%) mothers using BLW and 69 (86.3%) mothers not using BLW believe that the child can eat products from the family table, but they should be of suitable quality. The answer “yes, always” was chosen by 14 (5.8%) mothers practicing BLW and 5 (6.3%) mothers not using BLW. The calculated value of Cramer’s V coefficient for the assumed significance level α, *p* = 0.181 is 0.14 (strength of relationship weak). The observed differences in answers given by mothers using BLW and not using BLW are not statistically significant. The observed difference between the groups in the participants’ opinion that the child can eat products from the family table if the products are of the right quality is not significant, not only that the observed difference in responses was not statistically significant.

The analysis of the questionnaires showed that 231 women (231/320 = 72.2%), including 187 (77.9%) mothers using BLW and 44 (55.0%) not practicing BLW agreed with the statement that independent eating of soups by a child is one of the BLW methods. The calculated value of the Cramer’s V coefficient for the adopted level of significance is 0.22 (strength of relationship low). The observed differences in answers given by mothers using BLW and not using BLW are statistically significant.

For 300 (93.8%) of all mothers participating in the study, including 222 (92.5%) practicing the BLW method, the most important aim of dietary expansion with BLW is learning independent eating by the child. The second most important aim, indicated by 283 (88.4%) of all mothers, including 220 (91.7%) mothers who used the BLW method, was perfecting the skill of biting, chewing and swallowing food. A large part of mothers in total—223 (69.7%) of them, including 168 (70%) using BLW and 55 (68.8%) not using it, paid attention to the formation of self-regulation of the child’s appetite (Table 8).

## 4. Discussion

For many years, attention has been drawn to the fact that natural breastfeeding for newborns and infants is the gold standard in their nutrition. Mother’s milk is the most optimal food for babies during the first six months of life. Thanks to the ingredients contained in mothers’ milk, it contributes perfectly to the requirements of the baby’s growth and development [30,31]. The duration of breastfeeding in Poland has been assessed many times. Zagórecka et al. [32] found that at 6 months of age, 68.8% of children were still breastfed, and at 12 months of age. The study by Łukasik and Berek [33] showed that 57.1% of mothers breastfed their child naturally until 6 months of age, and after 6 months, this percentage decreased to 35.7%. The analysis of the study conducted by Fidler-Witoń et al. [34] shows that 85.5% of children aged 6 months were breastfed, but only breastfeeding was in 33.5% of the children studied, the rest of the children (52.0%) were fed with mother’s milk and milk formula. In the group of infants studied at 6 months of age by Mikiel-Kostyra et al. [35], only 13.2% of infants were exclusively breastfed. Frequent breastfeeding at the same age was reported by Stolarczyk et al. [36]; however, only 9% of infants were exclusively breastfed. Almost half of the children studied by Gawęda et al. [37] were breastfed for the first six months of life, but only 2.7% did not receive any additional products other than breast milk.

Over recent years, awareness of the importance of breastfeeding among infants has increased in Poland. As part of the National Health Programme, tasks are being implemented to support breastfeeding women. Actions carried out in the media and in pediatric clinics, such as the Week for the Promotion of Breastfeeding, are intended to increase awareness in this area [38]. According to data from the Central Statistical Office (Główny Urząd Statystyczny—GUS) for 2014, 98% of mothers breastfed after birth, at 6 weeks of age. 46%, between 2 and 6 months of age. 42%, at 9 months only 17%. However, the type and method of collecting these data make it impossible to compare them with results from other countries. This leaves us to rely on regional studies, although regional publications are characterized by a great diversity of presented results. Almost 97.7% of women start breastfeeding after birth, but only 50% do so exclusively; at 6 months of age, 38.6% continue to breastfeed, and the rate of exclusive breastfeeding during this period is only 3.7% [39,40]. Despite the solutions implemented by the National Health Program financed from the Polish state budget, there is a lack of obligatory training on lactation among medical personnel (physicians, midwives, and dieticians). Currently, in Poland, the education of pregnant and breastfeeding women is carried out exclusively by midwives (according to standards of perinatal care and antenatal schools), however, such activities should improve the current rates. At the same time, it is worth emphasizing that women from larger cities, with a higher level of education, more often take advantage of such educational solutions. Thus, depending on where the survey was conducted, the differences in breastfeeding duration in Poland are considerable [40,41].

The study by Marti-Solson E. et al. [42] used the PaPERC-BLW validated questionnaire assessing the parents’ perception of the BLW method; it demonstrated: the benefits of the BLW method in infant autonomy and development, and significant effects on child health; however, it was emphasized that the results of BLW studies must be evaluated using standardized validated questionnaires such as PaPerC-BLW, as studies concerning parents’ perception of BLW are difficult to generalize and should be treated with caution. Rowam H [43] analyzed food intake among BLW and traditionally eating (TW) children. The study showed that in infants aged 40–52 weeks, the mean intake in both groups was in line with WHO recommendations of 830 calories from milk and solid foods for infants aged 9–11 months. However, a study in a group of infants aged 26–39 weeks showed that the TW group consumed significantly more energy, carbohydrate, and protein, along with key micronutrients such as iron, calcium, and vitamin D. Pearce et al. [44] conducted among TW and BLW children at 6–8 months and 9–12 months observed that spoon-fed children (TW) regardless of age were fed more frequently. No differences in nutrient intake were observed among children aged 9–12 months. Considering complementary foods alone, only intakes of vitamin B12 and vitamin D were significantly higher in TW infants aged 6–8 months. Younger TW infants were more exposed to iron-fortified infant cereals and commercially produced infant foods. Quintiliano-Scarpelli et al. [45] emphasized that BLW is a relatively new complementary feeding practice from where further research is needed, including targeting health care professionals, and nutrition education is needed in this group as well as among parents.

Neves et al. [46] surveying health professionals in Brazil (65.3% dietitians), the majority of respondents fully agreed that the BLW method can be beneficial for children, in terms of sharing family meals, facilitating adaptation to food tastes and textures, improving chewing, and fostering the development of motor skills. However, important concerns have been highlighted regarding the convenience of BLW and the possibility of causing less fear or anxiety for parents. Arias-Ramos et al. [47] also analyzed the knowledge of health professionals about the BLW method. The study showed that health professionals considered the BLW method to facilitate family feeding, better adaptation to tastes and textures, influence chewing and motor skill development, and maybe a protective factor against obesity. However, they stressed that the BLW method is not recommended for all children.

Moore et al. [48] surveyed 3607 mothers and confirmed that knowledge of BLW guidelines is associated with exclusive breastfeeding for a longer period (regardless of demographic factors; *p* < 0.001); however, 80% of mothers stopped exclusive breastfeeding before the child reached 24 weeks of age and 65% before 17 weeks, despite knowledge of the guidelines. These data also support the findings of Brown et al. [6], which indicate that children who were fed using the BLW method were exclusively breastfed longer than children who were supplemented with traditional feeding (*p* < 0.001; 127.36 days for BLW versus 82.11 weeks for the traditional method). Despite the need for more research, these data suggest a potential longer breastfeeding duration among those using the BLW model. In our study, 318 (99.4%) of all mothers correctly considered that breastfeeding should not be abandoned when using BLW.

Over the past decade, many scientific papers and textbooks have been written on the use of the BLW method as a dietary expansion for infants. However, despite the revealed benefits associated with this method, health professionals are reluctant to advise the adoption of this new approach, especially given many concerns related to the possible negative effects on child health, increased risk of choking, and higher probability of low intake of energy and micronutrients, especially iron, as it is the child who decides about the quantity and quality of food, choosing from various options presented to him/her during meals [49,50,51]. It should be emphasized that also in the traditional method of dietary expansion presented by ESPGHAN and PTGHiŻD, it is the child who decides whether to eat and how much, and the parent who decides what to give the child [5,7].

In our study for 41.6% of women, including 36.7% of mothers using BLW the risk of choking was the most significant disadvantage. The risk of nutritional deficiency proved to be a significant disadvantage for 37.2% of them, including 43.8% of mothers not using BLW. A study [26] observed that an alarmingly high number of parents in all three groups (total BLW, partial BLW, and traditional dietary expansion) offered foods that were considered to pose a choking risk. Few studies report on the prevalence of choking. A study by Townsend E et al. found no difference in choking rates between the BLW and traditional dietary expansion groups [52]. In another study [53] of 199 infants fed according to BLW, 30% had at least one choking episode after eating solid food (apple). However, it cannot be excluded that such a high rate was caused by parents’ difficulties in distinguishing choking from gagging: Similar results were obtained by Brown et al. an observational study of 1151 infants on the risk of choking and whooping. The results of the study showed that at least one choking episode occurred in 11.9% of the group using BLW completely, in 15.5% in the group with partial use of BLW, and 11.6% in the group with traditional dietary expansion; the differences between the groups were not statistically significant [54].

The BLW method suggests several benefits, such as the prevention of obesity, as it takes into account self-regulation, a greater consumption of fruit and vegetables, better development of motor skills, and a positive influence on parental behavior. The child is encouraged to participate in family meals, without the pressure of time and amount of food consumed, and to interact with food, extensively exploring sensory aspects, by learning about different types of textures, and all this consequently creates a better relationship with food [55] In our study, 93.8% of mothers, including 92.5% of those practicing the BLW method, considered the most important aim of expanding the diet with BLW to be the learning of independent eating by the child. The second most important aim was the ability to improve biting, chewing, and swallowing food (88.4%), however, the majority of mothers were those who used BLW—91.7%. Additionally, a large part—69.7% (in the bow group used and does not use BLW) paid attention to the formation of self-regulation of a child’s appetite and 70.0% used BLW in practice.

Some authors suggest using the BLW method as the standard for complementary feeding because self-awareness of satiety and appetite contributes to healthy eating and behavioral patterns in the future [6]. Moore et al. conducted a study with 3607 participants who used the BLW method and found that 50% of mothers started complementary feeding before 23 weeks and 50% after 24 weeks. Starting complementary feeding at the right time was associated with greater knowledge of the guidelines for the BLW approach (*p* < 0.001) [48].

One of the critical points of approach to the BLW method is the lack of a formal definition and the use of different elements of this method at different times of dietary expansion, i.e., full BLW, partial BLW, and unconscious BLW. In its purest form, the BLW method should not include spoon-feeding, and the child should put food to the mouth independently [15]. However, in many definitions, BLW takes place when the proportion of purees and spoon-feeding during the day is less than 10% of the total food (BLW = 10% or less) [6]. According to the study by Cameron et al. infants fed according to the BLW principle are not spoon-fed at all, but they feed themselves whole pieces of food, preferably from the family meal, from the beginning of complementary feeding [53]. On the other hand, according to Brown et al. self-determination of parents whether the child eats using the BLW method is the same as using this method in the process of expanding the infant’s diet [54]. In our study mothers self-determined whether their children eat using the BLW method. Of the 320 mothers surveyed, 75.0% used the BLW method.

Summing up the above considerations we can say that there are many advantages and disadvantages of the BLW method, but in the light of scientific data, the balance is favorable. Therefore, a good understanding of the method is an important first step before starting its introduction in a child.

## 5. Conclusions

In the study group, mothers’ knowledge of the BLW method as a way of expanding a young child’s diet was insufficient. It seems important to implement appropriate educational activities on the methods of expanding children’s diets to broaden parents’ knowledge of the influence of nutrition on infant development.

In the study mothers who used the BLW method demonstrated better knowledge of expanding the diet of a young child with this method. It was found that mothers indicate the advantages and disadvantages of using the BLW method. This is an important result that indicates that the BLW method is not an ideal way of feeding; nevertheless, it is worth noting that the clear trend indicated in the cited studies supports its widespread use.

Mothers’ knowledge of the BLW method as a way of expanding a young child’s diet was insufficient in the study group. It seems important to implement appropriate educational activities on the methods of expanding children’s diets to broaden parents’ knowledge of the influence of nutrition on infant development.

## 6. Study Limitations

The results of our study should be interpreted, taking into account its limitations. Limitations of the study include the lack of diversity of the study group in terms of place of residence (mostly urban) and level of education (predominantly tertiary). All information was provided by mothers, which may cause information bias. Our study was a retrospective study, which may influence the occurrence of the false memory effect, especially in the group of mothers of older children aged 2–3 years, concerning details of infant dietary expansion.

In addition, the survey was conducted using the CAWI method, which is repeatedly criticized for its lack of insight into the data collection process, although it is worth noting that this type of data collection method is widely accepted and convenient for collecting large amounts of information in groups that are often difficult to access.

The advantage of the study is the size of the group of 320 mothers; so far, most of the studies on the use of the BLW method were carried out in smaller groups. It is also worth mentioning at this point that few studies on this topic have been conducted so far.

## Figures and Tables

**Table 1:** Characteristics of the study group of mothers.

	*n*	%
Age of mothers:		
Under 19 years	6	1.9%
19–30 years	169	52.8%
Over 30 years	145	45.3%
Place of residence:		
City	253	79.1%
Village	67	21.9%
Education:		
Higher	265	82.8%
Medium	50	15.6%
Vocational	3	0.9%
Basic	2	0.6%
Professional activity:		
Maternity/parental leave leave/sick	214	66.9%
Leave/unemployment		
White-collar work/studying	94	29.4%
Physical labor	12	4.7%
Childbearing:		
1 child in the family	222	69.4%
2 children in the family	89	27.8%
3 and more children in the family	9	2.8%
Age of child:		
0–4 months	19	5.9%
5–6 months	28	8.7%
7–12 months	114	35.6%
12–24 months	112	35.0%
24–36 months	47	14.7%

**Table 2:** Use of BLW in the surveyed group of mothers.

	*n*	%
Using BLW with your baby		
Yes	240	75.0%
No	80	25.0%
Reasons for not using the BLW method		
(*n* = 80; 25%):		
The child does not cooperate	30	37.5%
The child was not ready to start expanding the diet	20	25.0%
The BLW method is inappropriate for expanding a young child’s diet	15	18.75%
Lack of time to use BLW	7	8.75%
Use of BLW not recommended by mum/grandma/friends/others	5	6.25%
Mothers were concerned about the risk of their baby choking	3	3.75%

**Table 3:** Answers to the question “Should breastfeeding be abandoned while using the BLW method“—opinion among surveyed mothers.

	Giving up Breastfeeding While Using BLW					
	Yes, It Is Necessary		It Is Not Necessary		Total	
	*n*	%	*n*	%	*n*	%
Uses BLW	2	0.8%	238	99.2%	240	100%
Do not use BLW	0	0.0%	80	100%	80	100%
Total	2	0.6%	318	99.4%	320	100%

**Table 4:** Age of initiation of dietary expansion.

	Starting to Expand the Diet							
	4 Months–6 Months		6 Months		Above 6 Months of Age		Total	
	*n*	%	*n*	%	*n*	%	*n*	%
Uses BLW	7	2.9%	51	21.3%	182	75.8%	240	100%
Do not use BLW	4	5.0%	13	16.3%	63	75.8%	80	100%
Total	11	3.4%	64	20.0%	245	76.6%	320	100%

**Table 5:** Factors determining the start of dietary expansion with the BLW method among the surveyed mothers—multiple choice question.

The Deciding Factor in Starting to Expand the BLW Diet	Uses BLW		Do Not Use BLW		Total	
	*n*	%	*n*	%	*n*	%
Completed 4 months of age	6	2.5%	8	10.0%	14	4.4%
Completed 6 months of age	194	80.8%	53	66.3%	247	77.2%
Stable seating in chair/on lap	205	85.4%	65	81.3%	270	84.4%
Sitting alone	78	32.5%	20	25.0%	98	30.6%
Frequent awakenings during the night	1	0.4%	1	1.3%	2	0.6%
Doctor’s opinion	23	9.6%	21	26.3%	44	13.8%
Environment	0	0.0%	1	1.3%	1	0.3%
Opinion of mother/grandmother/family	3	1.3%	2	2.5%	5	1.6%
Total	240	100%	80	100%	320	100%

**Table 6:** Importance of BLW disadvantages in the group of mothers using BLW (*n* = 240).

	The Most Significant Disadvantage		Significant Flaw		Neither a Disadvantage nor an Advantage		In My Opinion, This Is Not a Disadvantage	
	*n*	%	*n*	%	*n*	%	*n*	%
Risk of choking	88	36.7%	73	30.4%	45	18.8%	34	14.2%
Risk of nutritional deficiencies	7	2.9%	84	35.0%	75	31.3%	74	30.8%
Unfavorable reactions from professionals/environment	3	1.3%	14	5.8%	75	21.3%	148	61.7%
Mess when learning to eat on your own	25	10.4%	57	23.8%	51	21.3%	107	44.9%
Problematic determination of the portion eaten by the child	8	3.3%	57	23.8%	58	24.2%	117	48.8%
The child decides how much and whether to eat a meal	6	2.5%	15	6.3%	40	16.7%	179	74.6%
The child associates meals with a form of play	6	2.5%	15	6.3%	75	21.3%	144	60.0%
Need to devote more time	7	2.9%	35	14.6%	65	27.1%	133	55.4%

**Table 7:** Importance of BLW disadvantages in the group of mothers not using BLW (*n* = 80).

	The Most Significant Disadvantage		Significant Flaw		Neither a Disadvantage nor an Advantage		In My Opinion, This Is Not a Disadvantage	
	*n*	%	*n*	%	*n*	%	*n*	%
Risk of choking	45	56.3%	23	28.8%	7	8.8%	5	6.3%
Risk of nutritional deficiencies	6	7.5%	35	43.8%	21	26.3%	18	22.5%
Unfavorable reactions from professionals/environment	0	0.0%	9	11.3%	30	37.5%	41	51.3%
Mess when learning to eat on your own	7	8.8%	22	27.5%	19	23.8%	32	40.0%
Problematic determination of the portion eaten by the child	6	7.5%	41	51.3%	18	22.5%	15	18.8%
The child decides how much and whether to eat a meal	0	0.0%	22	27.5%	17	21.3%	41	51.3%
The child associates meals with a form of play	8	10.0%	21	26.3%	24	30.0%	27	33.8%
Need to devote more time	9	11.3%	24	30.0%	25	31.3%	22	27.5%

**Table 8:** “The most important aim of expanding the diet with BLW” survey—multiple choice answers—figures do not add up to 100%.

The Most Important Aim of Expanding Your Diet with BLW	Uses BLW		Do Not Use BLW		Total	
	*n*	%	*n*	%	*n*	%
Weaning from milk	6	2.5%	1	1.3%	7	2.2%
Learning to eat on your own	222	92.5%	78	97.5%	300	93.8%
Making everyday life easier	33	13.8%	14	17.5%	47	14.7%
Improving the ability to chew, bite and swallow food	220	91.7%	63	78.8%	283	88.4%
To inspire confidence in the child	20	8.3%	4	5.0%	24	7.5%
Self-regulation of a child’s appetite	168	70.0%	55	68.8%	223	69.7%
Total	240	100%	80	100%	320	100%

## Data Availability

The data presented in this study are available on request from the corresponding author. The data are not publicly available due to restrictions that apply to the availability of these data.
